# Pattern of mother–child feeding interactions in preterm and term dyads at 18 and 24 months

**DOI:** 10.3389/fpsyg.2015.01245

**Published:** 2015-08-19

**Authors:** Paola Salvatori, Federica Andrei, Erica Neri, Ilaria Chirico, Elena Trombini

**Affiliations:** Department of Psychology, University of BolognaBologna, Italy

**Keywords:** prematurity, mother–child interaction, Feeding Scale, feeding and eating disorders of childhood, maternal depression

## Abstract

Literature on mother–child feeding interactions during the transition to self-feeding in preterm populations is lacking, particularly through observational methods. The present research study aims to look at the longitudinal patterns of mother–toddler feeding interactions, comparing preterm and full term dyads. To this end, a multi-method approach was used to collect data from 27 preterm to 20 full-term toddlers and their mothers. For each dyad, mother–child interactions were observed during the snack time at 18 and 24 months of age and then assessed through the Italian version of the Feeding Scale. Higher scores on the scale indicate a less healthy pattern of interaction. Additionally, at both points in time, mothers completed the *BDI-II* questionnaire as a screen for maternal depression and the child’s developmental stage was assessed using the Griffiths Scales. A series of repeated measures Analysis of Variances were run to detect differences in feeding interactions between the two groups at the time of assessment. Our results show that preterm dyads report overall higher levels of maternal negative affection, interactional conflicts, and less dyadic reciprocity during the meal compared to full-term dyads. Additionally, longitudinal data show that dyadic conflict decreases in both groups, whereas the child’s food refusal behaviors increase in the preterm group from 18 to 24 months. No differences were reported for both the BDI-II and the child’s development for the two groups. The results reveal that regardless of maternal depression and the child’s developmental stage, the two groups show different trajectories in the pattern of feeding interactions during the transition to self –feeding, at 18 and 24 months, with overall less positive interactions in preterm mother–child dyads.

## Introduction

Although transient eating difficulties are quite common in childhood and may be concurrent to life changes (Lindberg et al., 1991; Linscheid et al., 2009), it has been estimated that between 6 and 25–45% of children can experience eating disorders of various type and severity (Benoit, 2000; Lyons-Ruth et al., 2006; Bryant-Waugh et al., 2010). The risk and the complexity of eating problems are higher in the preterm born children population (Cerro et al., 2002; Pierrehumbert et al., 2003; Thoyre, 2007). These children are at risk for a number of developmental issues (Bhutta et al., 2002; Saigal and Doyle, 2008; Aarnoudse-Moens et al., 2009; McCormick et al., 2011) and nutrition has always represented a problematic area (Trombini, 2007). Preterm children might experience difficulties in the development of feeding skills, such as disorganized sucking patterns, failures in breastfeeding (Zanardo et al., 2011; Torola et al., 2012), and problems in swallowing semi-solids and solids (Mathisen et al., 2000; Burklow et al., 2002; Dodrill et al., 2004). Prematurity might also impact the long-term feeding behavior of the child and several studies have pointed out an increased risk of developing eating disorders in individuals born preterm (Cnattingius et al., 1999; Mathai et al., 2013; Vasylyeva et al., 2013; Micali et al., 2015). Although the association between prematurity and eating disorders in adulthood is still controversial (Krug et al., 2013), there is agreement that problems with feeding in childhood might persist into adulthood and affect other aspects of health, setting long-term risk for eating disorders, emotional, and behavioral problems (Marchi and Cohen, 1990; Ammaniti et al., 2012). Considering the importance of the parent-child relationship in the development of an healthy eating behavior (Satter, 1990; Chatoor, 1996), the Diagnostic Classification of Mental Health and Developmental Disorders of Infancy and Early Childhood (DC: Zero To Three, 2005) highlights the importance of the early detection of feeding problems, such as Feeding Disorder of State Regulation and Feeding Behavior Disorders (Chatoor, 1996, 2002, 2009), in vulnerable populations. Preterm infants might experience difficulties in the regulation of hunger-satiety cycles (Schädler et al., 2007; Schmid et al., 2011) and might require more help from their parents to maintain a state of calm alertness for feeding. Studies on preterm mother–infant feeding interaction show that premature infants seem to be less responsive to their mothers and to experience less clear interactions during feeding compared to full-term infants (Davis et al., 2003; Singer et al., 2003). In turn, the high levels of distress and depression described in mothers of preterm infants (Carter et al., 2007; Vigod et al., 2009; Voegtline et al., 2010; Brandon et al., 2011) might affect maternal capacity to detect and sensitively respond to the child’s cues during the interaction (Singer et al., 2003; Diego et al., 2006; Feldman and Eidelman, 2007; Agostini et al., 2014). Although findings are contradictory, several differences have been found in maternal interactive behaviors between full-term and preterm dyads (Bozzette, 2007; Korja et al., 2012). Mothers of preterm children have been described as more frequently overtly active and sometimes intrusive with their babies compared to mothers of full-term infants (Forcada-Guex et al., 2006; Feldman, 2007). Later, they seem to be less able to support the child’s autonomous play (Potharst et al., 2012). Moreover, mothers of preterm children seem to be more concerned about their child’s nutrition and weight gain than mothers of full-term children (Cnattingius et al., 1999; Cerro et al., 2002; Pierrehumbert et al., 2003), which in turn might influence maternal practices in feeding the child (Gueron-Sela et al., 2011). These contributions suggest that preterm mother–child dyads are at risk of experiencing early difficulties during feeding interactions. Feeding problems might occur as transactional or relational problems when the mother–child communication is impaired (Satter, 1990; Chatoor, 1996).

During the second year of life, as the child becomes increasingly physically and emotionally independent, the mother and the child must learn to negotiate a new reciprocal adjustment during feeding interactions (Lucarelli et al., 2003). The child shows the desire to feed him/herself and often rejects parent’s help during the meal (Freud, 1965; Morton et al., 1996; Cerro et al., 2002). The caregiver takes part in this process, creating an attuned relationship to support the child’s need for autonomy and his/her emotional-affective individuation (Spitz, 1957; Stern, 1985; Lichtenberg, 1989; Trombini and Trombini, 2007; Trombini, 2010). Failures in the mother–child communication might interfere with the transition to self-feeding and lead to highly conflictual interactions (Chatoor et al., 2000). For example, maternal excessive control and concerns related to the child’s seeking of autonomy (e.g., messiness, exploration with food, food preferences) might trigger distress in the child and lead to the child’s food refusal (Stein et al., 1999; Chatoor et al., 1998a, 2000; Trombini, 2010). Studies on mother–child feeding interactions in children with eating disorders have shown that conflicts are greater in these dyads compared to control dyads and are associated with maternal negative affect, low dyadic reciprocity, child’s food refusal, opposition, and negativism (Chatoor et al., 1998a; Lucarelli et al., 2003; Ammaniti et al., 2004; Atzaba-Poria et al., 2010).

The transition to self-feeding might be a critical time for the onset of eating disorders in childhood (Chatoor, 1996, 2002) and might present challenges for preterm mother–toddler dyads. Parental concerns for the child’s eating behavior may increase at this time (Morton et al., 1996; Cerro et al., 2002) and difficulties in supporting the child’s autonomy in mothers of preterm children (Potharst et al., 2012) may affect the quality of mother–child feeding interactions, interfering with the transition to self-feeding. Despite this, to our knowledge, there is a lack of investigations focused on the quality of mother–child feeding interactions during the second year of life in preterm populations.

The present study aims to address this gap by using a multi-method approach to investigate the longitudinal characteristics of feeding interactions in both preterm and full-term mother–child dyads. Assessments were conducted at two points in time, 18 and 24 months of age (corrected age for preterm children), a crucial time for the observation of the child’s developing eating autonomy. We expect the preterm group (PG) to show less positive mother–child feeding patterns compared to the full-term group and a higher rate of maternal and child’s dysfunctional behaviors during the meal (maternal negative affection, interactional conflicts, food refusal by the child, and low dyadic contingency). We also expect these interactive patterns to remain consistent from 18 to 24 months of age.

The second aim of the study is to evaluate the possible effect of both maternal depression and the child’s development on the quality of mother–child feeding interactions. With this regard, we expect the rate of maternal symptomatology to be higher in the PG, and preterm children to reach developmental milestones slower compared to full-term children.

## Materials and Methods

### Participants

The present study is part of a longitudinal project that involved preterm and full-term mother–child dyads from 18 to 30 months of the child. The participants comprised 47 mother–toddler dyads (27 preterm, 20 full-term).

The PG was recruited at the Neonatal Unit of the Bufalini Hospital in Cesena (Italy). All children enrolled in the follow-up program of the hospital and born with a gestational age (GA) ≤32 weeks and/or birth weight (BW) ≤1500 g were considered eligible for the study. Preterm toddlers and their mothers were consecutively recruited over the period March 2013–December 2014. Exclusion criteria were: (a) child’s major cerebral damage [intraventiricular haemorrhage (IVH) > III or IV grade, periventricular leucomalacia (PVL), retinopathy of prematurity (ROP), and hydrocephalus] or genetic syndrome; (b) parents’ past or present psychiatric history or the presence of neurological disorders; (c) parents’ past or present history of eating disorders (anorexia nervosa, bulimia nervosa, binge eating); (d) parent’s lack of proficiency in the Italian language. Among 38 dyads recruited, seven dyads were excluded from the sample as not matching the criteria for the study: one child reported cerebral damage (IVH > III grade); five dyads were excluded due to the parents’ lack of fluency in Italian, and one dyad due to the mother having a neurological disorder (multiple sclerosis). Four dyads dropped out after the first assessment. A total of 27 dyads were finally selected for the present research.

The mean GA of preterm children was 29.16 (SD = 1.99), and their mean BW was 1091.3 g (SD = 280.96). Fourteen children were males (51.9%) and 13 females (48.1%). The highest percentage of children was born with a cesarean (92.6%; *n* = 25) and only two with a spontaneous delivery (7.4%). Moreover, eight children (29.6%) were small for gestational age (SGA) and seven were twins (25.9%). All preterm mothers (*M*_age_ = 38.5, SD = 4.37 years) were employed, either married or cohabiting with the father of the child (88.5%), and most of them were Italian (85.2%). With regard to education, 47.8% had a high school diploma and 31.9% a university degree. Sixty-three percent of the mothers were primiparous at the time of the first assessment.

The group of full-term mother–child dyads (FG) was recruited from preschools in the area of Cesena (Italy) over the period April 2013–November 2014. As for the PG, exclusion criteria were: (a) child’s birth complications, cerebral damage, disabilities, or genetic syndromes; (b) parents’ past or present psychiatric history or the presence of neurological disorders; (c) parent’s past or present history of eating disorders; (d) parent’s lack of fluency in Italian. Twelve mothers declined their participation due to the inability to accommodate into the time schedule of the study. Twenty-one mothers accepted to take part in the project. Among these, one dyad was excluded from the sample due to major problems of the child (epilepsy). A final sample of 20 dyads was selected for the study.

All full-term children were born healthy, after 37 weeks of gestation (*M*_GA_ = 39.69, SD = 1.28), and with a BW over 2500 g (*M*_BW_ = 3455.25, SD = 462.79). Most children were born with a spontaneous delivery (60%). All mothers (*M*_age_ = 36.9, SD = 5.11 years) were married or cohabiting with the father of the child. Moreover, most of them were Italian (95%), employed (90%), primiparous (90%), and had a university degree (70%).

### Measures

#### Demographic and Obstetrical Variables

Relevant data on the child (e.g., GA, BW, past and recent clinical history) were collected from the infant’s medical records. Socio-demographic information about the mother (e.g., age, nationality, parity, level of education, marital status, occupation, past and present psychiatric and medical history, and occurrence of past or present eating disorders) was instead collected using an *ad hoc* designed questionnaire.

#### Mother–Child Feeding Interactions

The Feeding Scale (Chatoor et al., 1998b; Italian version SVIA-Scale di Sviluppo dell’Interazione Alimentare by Ammaniti et al., unpublished manuscript) was used to evaluate mother–child feeding interactions. The instrument is an observational scale, developed to be used from 0 to 36 months of age of the child, which allows the identification of the child’s and the mother’s dysfunctional behaviors during the meal through 46 items. The Italian version of the scale comprises four dimensions: Affective State of the Mother, Interactional Conflict, Food Refusal Behavior of the Child, and Affective State of the Dyad. Higher scores at each dimension indicate less healthy dyadic interactive patterns. The scale Affective State of the Mother measures the quality of maternal affect when feeding the child. High scores in this scale indicate a lack of pleasure and a prevalence of negative affect, such as sadness, anger, and distress. The scale Interactional Conflicts evaluates the presence of conflicts between the mother and the child during the meal. High scores in this scale indicate the presence of intrusive maternal behaviors (e.g., forcing the child to eat) while the child shows distress and avoidance during the feeding exchanges. The scale Food Refusal Behavior of the Child explores the characteristics of the child’s eating behavior and emotions during the meal. High scores on the scale indicate a high frequency of food refusal behaviors such as rejecting food, spitting, crying, negativity, and opposition. The scale also examines the presence of non-contingent maternal behaviors. Finally, the scale Affective State of the Dyads evaluates the quality of affect in mother–child relationship. High scores indicate a negative affective experience for the dyad and low dyadic reciprocity. The mother does not support the child’s autonomous initiatives, displaying controlling behaviors, insistent requests, and criticism, and the child responds showing distress and opposition.

The Feeding Scale showed good stability, inter-rater agreement and construct validity (Chatoor et al., 1997; Lucarelli et al., 2002). For the present study, Interclass Correlation Coefficient between the two raters ranged between 0.75 and 0.96 (mean = 0.90).

#### Child’s Level of Development

The child’s level of development was measured through the Griffiths Mental Development Scales (GMDS 0–2; Griffiths, 1996). The GMDS provides indication on the child’s mental and psychomotor development. Five areas are evaluated through the following subscales: Motor Development (54 items), Personal-Social (58 items), Hearing and Speech (56 items), Eye-Hand Coordination (54 items), Performance (54 items). A general quotient (GQ) score can also be computed from these five dimensions. Higher scores to each scale correspond to a superior development in a specific cognitive domain.

#### Maternal Depression

Maternal depression was evaluated with the Beck Depression Inventory (BDI-II; Beck et al., 1996; Italian version by Benvenuti et al., 1999). The BDI-II is a 21-item self-report, designed to assess the severity of depression in clinical and non-clinical populations. Each item is rated on a 4-point Likert scale ranging from 0 to 3; answers are given with reference to the previous 2 weeks. The BDI-II has high reliability and content validity, and it has shown to be effective in differentiating between clinical and non-clinical depression (Rickards et al., 2011).

In order to facilitate the analysis and to increase the sensitivity of the measure, for the purpose of the present study a general indicator of depressive symptoms was calculated as the average of the BDI-II scores between Time 1 (18 months after delivery) and Time 2 (24 months after delivery). This choice was supported by the high correlations (Cohen, 1988) between the two measurements both in the PG (*r* = 0.83, *p* < 0.001) and in the full-term group (*r* = 0.78, *p* < 0.01). Additionally, a cut-off score of 13 was used (low depression: 14–19; mild depression: 19–29; severe depression: 30–63; Beck et al., 1996).

### Procedure

After approval was obtained by the ethic committee of the Department of Psychology, and informed written consent was signed by participants, all dyads were assessed at the Psychodynamic Research Laboratory “Anna Martini” of the University of Bologna (Cesena, Italy). The assessments were conducted at 18 and 24 months of the child (corrected age for preterm children) through the same multi-method procedure.

First, the feeding session was observed during the morning/afternoon snack time. Observations were scheduled in agreement with each mother in order to respect their child’s eating habits. Prior to the assessment, mothers were instructed to bring the child’s usual snack and to behave as they would normally do at home. Twenty minutes of feeding interaction were videotaped from behind a one-way mirror and later coded by two raters, blind to the child’s condition, through the Feeding Scale. Secondly, a psychologist measured the child’s level of development through the GMDS. Last, mothers were asked to complete the BDI-II.

### Statistical Analysis

Differences between full-term and preterm mothers in demographic and obstetric variables, as well as differences between full-term and preterm children’s level of development were investigated through a series of Chi-Squares and Student’s *t*-tests. Repeated measures Analysis of Variance (ANOVA) was also used to evaluate differences in depression symptomatology between preterm and full-terms mothers at each time of assessment.

Pearson’s correlation coefficients were calculated to test bivariate associations between the BDI-II and the Feeding Scales dimensions scores in each group at both Times 1 and 2. For each dimension of the Feeding Scale a within-between repeated measures ANOVA was run to test between groups (pre- vs. full-term dyads) differences in feeding interactions by time of assessment (18 and 24 months). Where a significant correlation emerged between the Feeding Scales and scores on the BDI, the latter were added as covariate to the model. Greenhouse–Geisser epsilon adjustment to the degrees of freedom was performed, when appropriate, and adjusted *p*-values are reported. In order to further analyze significant effects, Bonferroni correction for multiple comparisons was employed.

## Results

### Maternal Variables

Demographic characteristics of the study sample are displayed in **Table 1**. No significant differences emerged with the exception of parity [*X*^2^(1,47) = 4.42, *p* < 0.05], as the percentage of multiparous women was significantly higher in the pre-term group (37%) than in the full-term group (10%).

**Table 1:** Socio-demographic characteristics of the sample.

	Preterm group (PG; *N* = 27)	Full-term group (*N* = 20)	*t*/*X*^2^
**Child**			
Gender, male, *n* (%)	14 (51.9%)	14 (70%)	1.571
Birth weight, mean ± SD	1091 ± 280.96	3455 ± 462.79	20.246^∗∗∗^
Gestational age, mean ± SD	29.16 ± 1.99	39.69 ± 1.28	20.631^∗∗∗^
Small for gestational age (SGA), *n* (%)	8 (29.6%)	0 (0%)	7.142^∗∗^
Type of delivery, *n* (%)			15.195^∗∗∗^
Spontaneous	2 (7.4%)	12 (60%)	
Cesarean	25 (92.6%)	8 (40%)	
Twins, *n* (%)	7 (25.9%)	0 (0%)	6.093^∗^
**Mother**			
Mean age (in years), mean ± SD	38.5 ± 4.46	36.9 ± 5.15	1.146
Nationality, *n* (%)			1.164
Italian	23 (85.2%)	19 (95%)	
Foreign	4 (14.8%)	1 (5%)	
Civil status			2.469
Married or cohabiting	24 (88.5%)	20 (100%)	
Single	3 (11.5%)	0 (0%)	
Education, *n* (%)			4.366
Middle school certificate	3 (13%)	2 (10%)	
High school diploma	12 (47.8%)	4 (20%)	
University degree	12 (31.9%)	14 (70%)	
Occupation, *n* (%)			2.820
Employed	27 (100%)	18 (90%)	
Unemployed	0 (0%)	2 (10%)	
Parity, *n* (%)			4.417^∗^
Primiparous	17 (63%)	18 (90%)	
Multiparous	10 (37%)	2 (10%)	

*^∗^*p* < 0.05, ^∗∗^*p* < 0.01, ^∗∗∗^*p* < 0.001.*

Regarding maternal depression levels, no significant differences were observed for Group [*F*(1,42) = 1.82, *p* = 0.18, $\eta_{p}^{2}$ = 0.04], Time [*F*(1,42) = 0.09, *p* = 0.77, $\eta_{p}^{2}$ = 0.002] and Time × Group variables [*F*(1,42) = 2.43, *p* = 0.12, $\eta_{p}^{2}$ = 0.05; see **Table 2**]. Maternal mean scores on the BDI-II for the full term group were 8.74 ± 6.91 at 18 months and 8.05 ± 6.90 at 24 months. PG scores were 6.67 ± 4.85 at 18 months and 5.60 ± 5.35 at 24 months.

**Table 2:** Mean scores and SD of the Feeding Scale at 18 and 24 months.

Measures	PG		Full-term group	
	18 months *M* ± SD	24 months *M* ± SD	18 months *M* ± SD	24 months *M* ± SD
Affective State of the Mother	8.33 ± 4.02	8.70 ± 3.79	5.40 ± 2.56	3.90 ± 2.69
Interactional Conflicts	9.19 ± 5.51	8.67 ± 4.82	7.85 ± 4.33	4.70 ± 2.40
Food Refusal Behavior of the Child	1.70 ± 1.61	2.30 ± 1.46	1.95 ± 2.18	1.30 ± 1.49
Affective State of the Dyad	2.56 ± 2.10	2.96 ± 1.60	2.15 ± 1.30	1.55 ± 1.53

### Child’s Variables

Differences were observed between the two groups for children’s weight and GA, and type of delivery (see **Table 1**), whereas no differences emerged for the child’s gender [*X*^2^(1,47) = 1.57, *p* = 0.21]. Results from *t*-test showed no differences in children’s GQ levels measured through the Griffiths Scale at 18 months [*t*(1,45) = -0.93, *p* = 0.36] and at 24 months [*t*(1,44) = -1.84, *p* = 0.07]. At 18 months, the mean GQ score was 101.59 ± 9.84 in the PG (*N* = 27) and 104.10 ± 7.98 in the control group (*N* = 20). At 24 months, the mean GQ score was 103.38 ± 10.32 in the PG (*N* = 26) and 108.45 ± 7.64 in the control group (*N* = 20). As no differences emerged in the child’s level of development, this variable was not taken into account in subsequent analyses.

### Bivariate Relationships Among Study Measures

Pearson’s coefficients indicated that scores on the BDI-II were positively correlated to the subscale Affective State of the Dyads at 18 (*r* = 0.45, *p* = 0.01) and, although marginally significant, at 24 months (*r* = 0.39, *p* = 0.04) in the PG. When checking for the control group no significant correlation emerged between symptoms of depression and scores on the subscales of the Feeding Scale at 18 and 24 months (all *p*s > 0.05).

### Mother–Child Feeding Interaction

**FIGURE 1:**
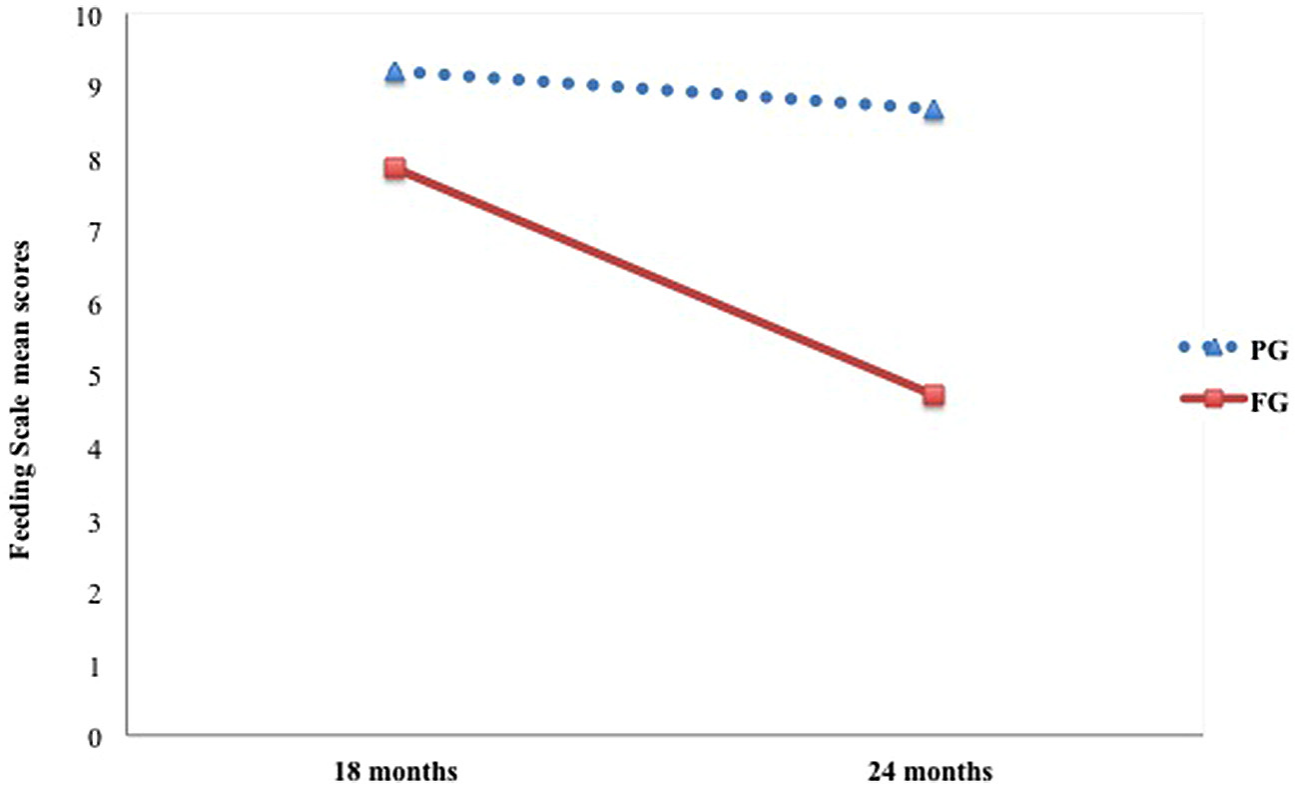
**Longitudinal trajectories in preterm and full-term dyads for the scale Interactional Conflict**.

**FIGURE 2:**
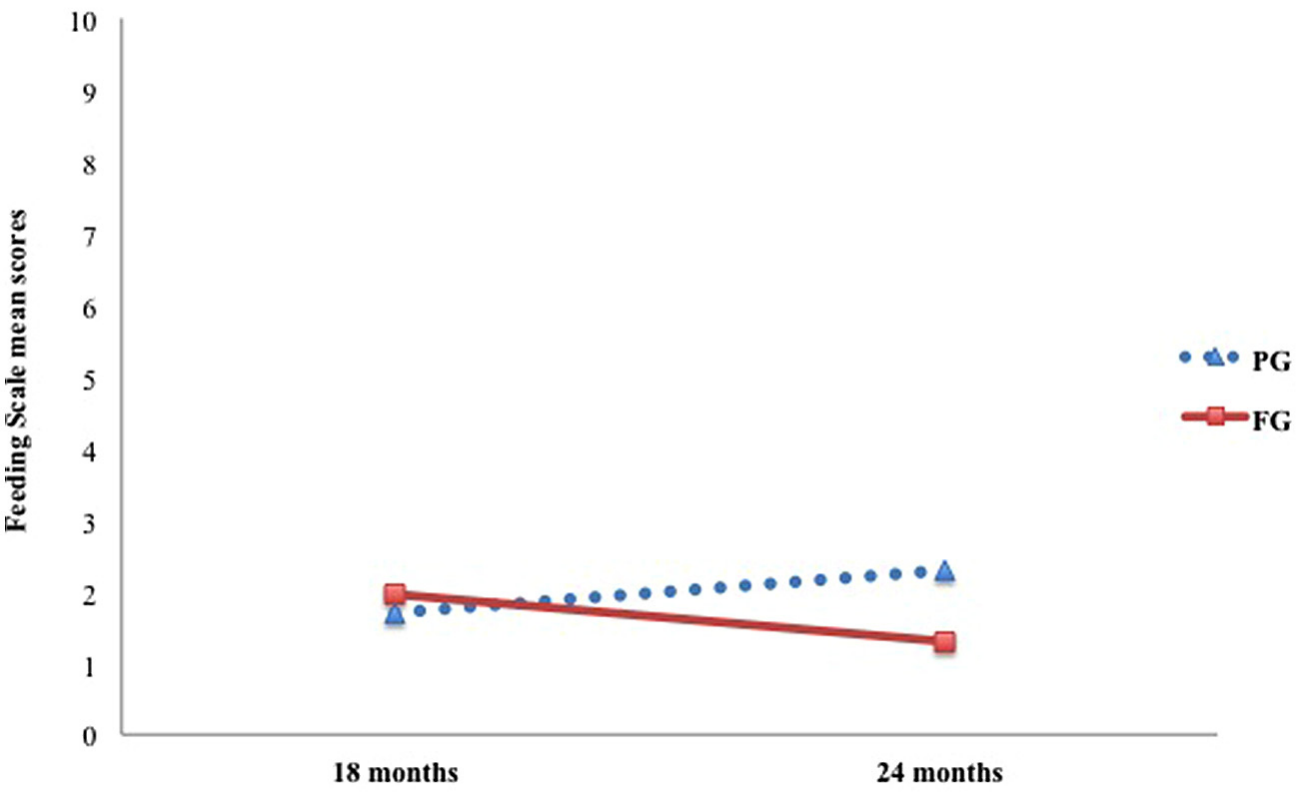
**Longitudinal trajectories in preterm and full-term dyads for the scale Food Refusal Behavior of the Child**.

**Table 2** shows mean scores on the Feeding Scale for each group. Results for the dimension Affective State of the Mother revealed a significant main effect of group only [*F*(1,45) = 20.35, *p* < 0.001, $\eta_{p}^{2}$ = 0.31], with mothers from the pre-term group scoring higher than mothers from the full-term group (*p* < 0.001). Regarding the dimension Interactional Conflict, main effects of group [*F*(1,45) = 5.66, *p* < 0.05, $\eta_{p}^{2}$ = 0.11] and time of assessment [*F*(1,45) = 6.06, *p* < 0.05, $\eta_{p}^{2}$ = 0.12] were detected. In this case, for both groups a significant decrease in Interactional Conflict scores from 18 to 24 months emerged (*p* < 0.05). However, the pre-term group reached higher scores at this dimension than the full-term group (*p* < 0.05), thus implying overall greater levels of interactional conflict between pre-term children and their mothers compared to full-term dyads (**Figure 1**). With respect to Food Refusal, only the interaction Time × Group was significant [*F*(1,45) = 7.32, *p* = 0.01, $\eta_{p}^{2}$ = 0.14]. Particularly, at 24 months preterm children showed significantly higher levels of food refusal than at 18 months compared to full-term children, whose scores remained lower and almost unvaried between the two assessments (**Figure 2**).

Given that in the pre-term group a significant correlation between levels of maternal depression and Affective State of the Dyads was found, for this dimension of the Feeding Scale mean scores at the BDI were considered as covariate. After controlling for the effects of depression, results showed a significant main effect of group [*F*(1,45) = 8.01, *p* < 0.01, $\eta_{p}^{2}$ = 0.15], and mean difference revealed an overall worse affective state of pre-term compared to full-term dyads.

## Discussion

The aim of this study was to explore the characteristics of mother–toddler feeding interactions between 18 and 24 months of age, comparing preterm with full-term dyads. Maternal depression and the child’s developmental stage at both assessments were considered as additional influential variables.

Results partly confirmed our hypothesis. Main differences between groups emerged in three out of four dimensions of the Feeding Scale. Preterm mother–child dyads obtained significantly higher scores on the subscales concerning the mother (i.e., Affective State of the Mother), and the dyad (i.e., Interactional Conflict and Affective State of the Dyad), compared to full-term mother–child dyads. Group mean scores pertaining to the ‘Affective State of the Mother’ indicate that, compared to the full-term group, mothers of preterm children show more angriness, sadness and distress and less pleasure when feeding their child. Dyadic measures on the scales ‘Interactional Conflict’ and ‘Affective state of the Dyad’ indicate the presence of more frequent and intense dyadic conflicts, lack of reciprocity, and negative affect in preterm dyads than in full term ones. Specifically, our findings reveal that preterm mothers are more intrusive and controlling during feeding, and they support the child’s autonomous initiatives less than full-term mothers. In turn, preterm toddlers show higher distress, avoidance and negativity compared to full-term children. This result indicates that preterm mother–toddler dyads, compared to full-term ones, show a higher risk of experiencing overall less positive and less contingent interactions during the meal. Consistent with our results, other studies have documented less contingent feeding interactions in preterm than in full-term dyads (Davis et al., 2003; Singer et al., 2003). Particularly, previous findings show that in the early postpartum period, preterm infants are less responsive to maternal cues during feeding compared to full-term babies (Davis et al., 2003; Singer et al., 2003). Singer et al. (2003) also suggest that maternal high levels of distress affect the mother’s capacity to contingently and sensitively respond to the child’s cues during feeding. Our findings seem to confirm these results, showing that a lack of dyadic contingency during feeding interactions might persist over time in preterm mother–toddler dyads.

Chatoor et al.’s (1998a; 2000) has highlighted that interactive conflicts during the meal and maternal difficulties in sensitively and contingently responding to their toddlers’ feeding behavior can interfere with the successful transition to self-feeding and lead to feeding disorders through several pathways. Current research shows that both maternal psychopathology and the child’s challenging temperament and behavior may hinder the creation of dyadic contingency during the interaction, leading to chronic mismatches between the toddlers’ behavior and the parent’s response, thus fostering negative affect and conflicts during feeding (Chatoor et al., 2000; Feldman et al., 2004; Ammaniti et al., 2010). It must be acknowledged that preterm children are at risk for regulatory, emotional, and behavioral problems such as negative mood, irritability, distractibility, and low tolerance to stimuli (Langkamp and Pascoe, 2001; Hughes et al., 2002; Weiss et al., 2004; Klein et al., 2013). Moreover, many preterm children suffered trauma related to medical intervention and invasive procedures (i.e., endotracheal suctioning, intubation, nasogastric feeding tube) and might report gastro-esophageal reflux, oral-hypersensitivity or oral-motor difficulties (Dodrill et al., 2004; Torola et al., 2012). These factors might influence the child’s attitude toward eating and lead to intense fear of food, food aversions, lack of hunger, or appetite (Chatoor, 2002). In turn, there is evidence of the association between children’s eating behavior and their mothers’ perception and feeding practices (Ramsay and Gisel, 1996; Chatoor et al., 2000). Therefore, the child’s clinical history, his/her temperamental and behavioral characteristics might have influenced the pattern of feeding that we observed in preterm dyads. For this reason, the role of these variables should be explored in future studies.

Regarding the stability of the interactive feeding patterns observed over time, we found that the scores on the ‘Affective State of the Mother’ and ‘Affective State of the Dyad’ scales were fairly stable from the first to the second assessment. Contrarily, the time factor led to differences between the two groups with respect to the scale ‘Interactional Conflict’. The scores of both groups in this dimension dropped from 18 to 24 months, indicating a progressive decrease in dyadic conflict levels. According to the Feeding Scale validation studies, the ‘Interactional Conflict’ scale score normally increases between 9–12 and 12–18 months of age, as the child starts spoon- and self-feeding, followed by a gradual decline in that score from 18 to 24 months of age onwards (Chatoor et al., 1997; Lucarelli et al., 2002). Our results seem to reflect a similar trend. Although mean scores of the PG remained significantly higher than those of the full-term group, the decrease of conflict in preterm dyads suggests an improvement in mother–child interaction. All preterm dyads participating in the study were involved in a follow up program aimed at monitoring and supporting the child’s development from the time of discharge till they reached 30 months of age. This program included the provision of psychological and educational support to parents to promote their awareness and involvement in the child’s care. The improvement observed could be influenced by the psychological support provided to the parents and to the children.

However, time also seemed to play a role in differences between the two groups on the ‘Food Refusal Behavior’ Scale. Scores of the PG significantly increased from 18 to 24 months, indicating a greater frequency and intensity of the child’s protests and food avoidance behaviors. Differently, scores of the full-term group remained fairly low and stable over time. Literature shows that, usually, in healthy full-term dyads, dyadic reciprocity and maternal contingency increase, whereas dyadic conflict and food refusal behaviors gradually decrease from the first to the second year of life, reflecting an overall adjustment of feeding interactions as most of the issues related to autonomy are overcome (Lucarelli et al., 2003; Ammaniti et al., 2004). In our sample, the longitudinal trajectories observed in the PG seem to reflect a different trend. Even though dyadic conflict decreased, dyadic reciprocity, and maternal contingency remained low, and the child’s food refusal behaviors increased. Hence, further longitudinal studies are needed to investigate the development of these interactive patterns over time and their impact on the child’s subsequent eating behavior.

In our sample, the child’s developmental level and the presence of maternal depression had no effect on the quality of mother–child feeding interactions, and this disconfirmed our second hypothesis. Additionally, our results showed that there were no significant differences between preterm and full-term toddlers’ GQ scores at 18 and 24 months and that both groups were developing normally. These findings diverge from those of previous studies reporting lower GQ scores in preterm children compared to full-term children (Bhutta et al., 2002). However, several studies also highlight a broad inter-individual variability related to the severity of prematurity (i.e., extreme BW and GA, obstetric complication), which might increase the risk of negative neurodevelopmental outcomes (Marlow et al., 2005; Gianni et al., 2007; Claas et al., 2011; Sansavini et al., 2011; Stoinska and Gadzinowski, 2011; Biasini et al., 2015). These variables, which may intensify the differences observed between full term and preterm children, were not taken into account in the present study due to the small sample size. Moreover, our results could be partially explained considering the influential role of the follow-up intervention carried on with preterm children and their parents. Indeed, literature evidences the effect of similar interventions in promoting the child’s positive cognitive development during early and middle childhood (Kaaresen et al., 2006; Melnyk et al., 2006; Orton et al., 2009).

Regarding the presence of maternal depression, our findings did not show any significant difference between pre- and full-term mothers. For both groups medium scores on the BDI were below clinically relevant levels, thus not providing support to our hypothesis. Most of the studies that detected a high risk of depression in mothers of preterm children have been conducted in the first year postpartum (Carter et al., 2007; Vigod et al., 2009; Voegtline et al., 2010) and less is known on the evolution of the symptomatology over time. Miles et al. (2007) found that levels of depression in preterm children’s mothers tend to decrease from the first month post-partum to 6 months and then remain stable. Similar results were obtained by Singer et al. (1999), who found that distress levels for pre-term mothers decreased from the early postpartum to 3 years of age, thus reaching the same levels as mothers of full-term children. Our findings seem to support the hypothesis that the prevalence of maternal depression in the second year of the child’s life may be less influenced by prematurity than in the early post-partum period. However, it must be noted that mothers participating in our research were mainly wealthy and educated women, married, or cohabiting with the father of the child. Previous studies show that the remission of depressive symptoms over time is lower in the presence of concurrent risk factors concerning the child, the mother, as well as the family (Poehlmann et al., 2009). Moreover, the support provided to parents of preterm children’s might have promoted positive outcomes for the mothers’ mental health, as well as for the child’s cognitive development (Melnyk et al., 2006; Trombini et al., 2008; Montirosso et al., 2012; Weber et al., 2012). Future studies should control for the effects of psychological intervention with mothers of preterm children.

Last, a significant correlation between maternal depression and the dimension ‘Affective State of the Dyad’ of the Feeding Scale was found in the PG. High scores in this scale indicate the dyad experience negative affective involvement, resulting from maternal difficulties in supporting the child’s autonomous initiatives (controlling behaviors and criticism) and child’s responses of distress and negativism. This result suggests an association between a greater depressive state and a diminished maternal capacity to detect and sensitively respond to the child’s cues during the interaction, confirming previous studies (Singer et al., 2003; Diego et al., 2006; Feldman and Eidelman, 2007; Agostini et al., 2014). However, our data show no significant correlations in the full-term group. Hence, the association between depression and negative affective state of the dyad during feeding seems stronger when maternal depression and the child’s prematurity co-occur. This result suggests the clinical relevance of monitoring maternal mental state in preterm dyads.

Some limitations of the study must be considered when interpreting our findings. First, due to the small sample size, all results should be replicated on larger samples. Second, we did not measure some characteristics of the child, such as temperamental difficulties and behavioral problems, which might have played a role on the pattern of feeding observed. Moreover, we lack data on the clinical history of preterm children, such as the presence of major aversive event or repeated noxious insults to the oropharynx or gastrointestinal tract (e.g., reflux, insertion of nasogastric feeding tube or endotracheal suctioning), which trigger intense distress in the infant or young child and might affect the pattern of eating (Chatoor, 2002). Last, the literature shows that maternal attitudes toward food and concerns about the child’s behavior might influence her feeding practices (Agras et al., 1999; Stein et al., 1999; Gueron-Sela et al., 2011). Hence, these elements pertaining both preterm children and their caregivers should be taken into account by future investigations. Indeed, feeding interactions are part of the general relationship between the mother and the child, which is influenced by both parties’ characteristics and histories (Satter, 1990; Chatoor, 1996). Taking into account these aspects and considering that the eating pattern in preterm dyads is the result of many combined factors, future studies should be directed to better evaluate the effect of the child’s and the mother’s characteristics on the pattern of feeding interactions observed in preterm dyads.

Despite some limitations, our findings show that preterm mother–toddler dyads experience less positive interactions during the transition to self-feeding compared to full-term mother–toddler dyads, displaying more maternal negative affect, dyadic conflicts and lack of reciprocity during the meal. Future data from the longitudinal study will allow a more thorough understanding of the evolution of the interactive patterns observed at 18 and 24 months over time and the related risk on the child’s subsequent eating attitudes and behavior.

## Conflict of Interest Statement

The authors declare that the research was conducted in the absence of any commercial or financial relationships that could be construed as a potential conflict of interest.
